# Pretreatment drug resistance profiles and transmission dynamics of HIV-1 among adults aged ≥50 in Hebei Province, China

**DOI:** 10.3389/fcimb.2026.1823851

**Published:** 2026-05-13

**Authors:** Xinyue Fan, Meng Liu, Ning An, Guangyi Bai, Xueang Xu, Meiqi Meng, Lihua Cui, Qi Li, Xinli Lu

**Affiliations:** 1 School of Public Health, North China University of Science and Technology, Tangshan, Hebei, China; 2 Department of AIDS Research, Hebei Key Laboratory of Pathogen and Epidemiology of Infectious Disease, Hebei Provincial Center for Disease Control and Prevention, Shijiazhuang, Hebei, China

**Keywords:** HIV-1, marital status, molecular epidemiology, molecular transmission network, older adults, phylogenetic analysis, pretreatment drug resistance, viral subtype

## Abstract

**Background:**

In China, the proportion of HIV-infected individuals aged ≥50 is rapidly increasing, yet limited research has focused on HIV transmission dynamics in this population. We investigated pretreatment drug resistance (PDR) and molecular transmission networks among older adults with HIV-1 in Hebei Province.

**Methods:**

From 2022 to 2025, 334 newly confirmed, ART-naïve HIV-1-infected individuals aged ≥50 with successful *pol* gene amplification were included. HIV-1 *pol* gene sequencing was performed, with drug resistance mutations and phenotypes determined via the Stanford HIV Drug Resistance Database. A molecular transmission network was constructed using a 1.5% genetic distance threshold.

**Results:**

The participants were predominantly male (80.2%), with contact between men who have sex with men (MSM) as the main transmission route (65.3%). CRF07_BC (42.5%) and CRF01_AE (38.0%) were dominant. The overall PDR prevalence was 12.3%, with non-nucleoside reverse transcriptase inhibitor (NNRTI) resistance being the most common (6.6%). The clustering rate was 35.0%. Multivariate analysis identified divorced/widowed status (adjusted odds ratio [aOR] = 2.040), residence in Eastern Hebei (aOR = 2.401), and CRF07_BC infection as independent risk factors for cluster inclusion. Notably, 77.8% of the participants were married, indicating potential spousal transmission through bisexual behavior.

**Conclusion:**

MSM is the predominant transmission route among older adults in Hebei. Marital dissolution drives active transmission cluster involvement, while the high proportion of married individuals underscores spousal transmission risk. Given the high PDR prevalence and specific vulnerabilities of older adults, transitioning to integrase strand transfer inhibitor (INSTI)-based regimens and targeted spousal interventions are urgent priorities.

## Introduction

Although antiretroviral therapy (ART) has made acquired immunodeficiency syndrome (AIDS) a manageable chronic condition, the human immunodeficiency virus (HIV) continues to pose a significant global public health threat, and the demographic characteristics have changed significantly in recent years (Joint United Nations Programme on HIV/AIDS, 2023). In China, the demographic structure of the AIDS epidemic is also changing rapidly, the most prominent manifestation of which is the accelerated “aging” of HIV-infected groups. Recently, the new infection rate of older adults (aged ≥50) has continued to rise, presenting a critical challenge for current prevention and control work (Huang et al., 2024). According to the model forecast data (Zhai et al., 2024), if there is a lack of targeted interventions, the disease burden of this age group will continue to increase in the next decade and is expected to exceed that of the young population.

Older adults often lack awareness of safe sex and self-protection. In addition, “empty nest” syndrome causes unmet emotional and physical needs, which may lead to unprotected sexual contact and subsequent infection (Huang et al., 2024). Exacerbating this risk, older adults are often diagnosed in the later stage of infection, which usually complicates their treatment, results in slower immune recovery, and is accompanied by chronic diseases. Therefore, precise strategies tailored to the specific needs of this population are urgently needed (Yu et al., 2022). Traditional epidemiological investigation methods have limited ability to identify hidden transmission chains. Molecular transmission network analysis based on viral gene sequencing addresses this limitation by serving as a key tool to define transmission clusters and accurately target high-risk transmission nodes (Brenner et al., 2021). Furthermore, the appearance of drug resistance before treatment is compromising the efficacy of the treatment plan. If this issue is not addressed, it will likely worsen the spread of drug-resistant strains. This, in turn, will significantly hinder the progress toward the UNAIDS “95-95-95” targets (Joint United Nations Programme on HIV/AIDS, 2023). Previous studies have described the HIV epidemic in eastern and southern China in detail (Yuan et al., 2023). In contrast, comprehensive epidemiological data on HIV infection among older adults in Hebei Province—a populous province in northern China—remain notably absent. To address this critical knowledge gap, the present study integrates pretreatment drug resistance (PDR) profiling with molecular transmission network analysis. These two approaches are examined together precisely because they feed into each other in the context of epidemic control. Profiling PDR establishes the baseline risk of treatment failure at the individual level, while molecular networks reveal the active transmission chains that push resistant strains outward across the population. By looking at them side by side, this study seeks to go beyond a simple tally of drug resistance prevalence. It aims to trace the actual routes of spread, laying down a solid empirical base for designing regional intervention strategies that are both precise and locally tailored. The epidemiological data from Hebei Province point to a demographic shift that is gathering pace and reshaping the local HIV burden. Between 2016 and 2022, adults aged ≥50 made up roughly 24.9% (16.0% for ages 50–59 and 8.9% for ages ≥60) of all newly reported HIV/AIDS cases in the province (Wu et al., 2024). The scale and persistent upward trend of this share make a strong case for studies that zero in on this older segment of the population. Monitoring PDR offers a practical way to pick up both transmitted resistance and any unnoticed prior antiretroviral use, which, in turn, yields a more realistic gauge of the clinical vulnerabilities faced by older adults.

## Materials and methods

### Study participants and data collection

From January 1, 2022, to June 1, 2025, we enrolled newly confirmed HIV-1-positive individuals aged ≥50 from the Hebei Provincial AIDS Confirmation Center, all of whom had never received antiretroviral therapy. These individuals were identified primarily through passive routine screening and voluntary counseling; no active contact tracing was involved. While active tracing typically yields higher clustering rates due to the presence of known epidemiological links, our reliance on passive detection brings into sharp relief transmission networks that had previously gone unnoticed. A total of 376 samples were collected in this study, and demographic data, treatment history, and follow-up information (including self-reported transmission routes) of the corresponding subjects were extracted directly from China’s National Comprehensive Response Information Management System for HIV/AIDS, supplemented by epidemiological field investigation forms routinely administered by the local Center for Disease Control and Prevention. We stratified Hebei’s study area by administrative divisions into four regions: Eastern, Central, Southern, and Northern Hebei. Written informed consent was obtained from all participants prior to enrollment. From each participant, we extracted 5 mL of blood from the peripheral vein and collected it using EDTA anticoagulant tubes. Within 6 h after collection, the sample was centrifuged at 1,500 × g for 10 min to separate the plasma. The plasma was then divided and frozen at -80°C for subsequent analysis. This study was approved by the Ethics Committee of the Hebei Provincial Center for Disease Control and Prevention (approval number: HeBCDCIRB (S) 2021-029).

### RNA extraction, amplification, and sequencing

Viral RNA isolation was performed on the EXM 600 automated platform (Zybio Inc., Chongqing, China). For this procedure, we utilized the compatible Zybio extraction kit (number 2402007) in accordance with standard protocols. According to our previously reported method (Lu et al., 2017), gene analysis was carried out for the protease and reverse transcriptase coding region (*HXB2*: 2550–3870) of the HIV-1 *pol* gene. The 1.3-kb fragment was amplified by one-step RT-PCR, followed by a second round of nested PCR, and the reaction mixture was prepared using the PrimeScript™ One Step RT-PCR Kit Ver. 2 (Takara Bio Inc., Japan). The final amplified PCR product was submitted to Beijing BMD Sequencing Co., Ltd., to complete bidirectional Sanger sequencing.

### Sequence analysis and HIV-1 subtype determination

This study used Sequencher software (version 5.4.4) to assemble the original sequencing data to obtain consensus sequences. The sequences were subsequently aligned against reference strains using the HIV-Align online tool (Los Alamos National Laboratory HIV Database, https://www.hiv.lanl.gov/). The alignment results were submitted to the Stanford University HIV Drug Resistance Database, and the HIVdb program was used for genotype determination and drug resistance analysis. To verify the reliability of the results, we also performed a phylogenetic analysis using MEGA 11.0. In individual cases where the database inference result was inconsistent with the topology of the phylogenetic tree, the final genotype of the sample was further confirmed by HIV BLAST comparison.

### Analysis of drug resistance mutations

This research used the HIV Drug Resistance Database from Stanford University (https://hivdb.stanford.edu/) to evaluate viral resistance to three main classes of antiretroviral drugs: nucleoside reverse transcriptase inhibitors, non-nucleoside reverse transcriptase inhibitors, and protease inhibitors. The analysis included all 25 drugs available in the database. The degree of drug resistance was classified into five levels according to the standard algorithm: susceptible (S), potential (P), low-level (L), intermediate (I), and high-level (H). If low-level or higher resistance to at least one drug of a certain class was present, it was defined as resistance to that category.

### Molecular transmission network construction

After completing pairwise genetic distance calculations based on the TN93 model in the HyPhy platform, Sequencher software (version 5.4.4) was further used for analysis (Zhang et al., 2023). To achieve a balance between the number and scale of transmission clusters, we systematically evaluated thresholds within the range of 0.1%–2.0% (Supplementary Material 1). Based on the results of the sensitivity analysis, 1.5% was ultimately selected as the genetic distance threshold to construct the molecular transmission network, and network visualization was performed using Cytoscape 3.7.2. In the network, the node “degree” is an important metric. Its value reflects the centrality of the node within the network, which typically implies that the node may have strong transmission potential (Chen et al., 2022). According to the HIV Transmission Network Surveillance and Intervention Technical Guidelines, this study classifies individuals with a degree value ≥4 as high-risk transmission cases (Zhang et al., 2023). Additionally, connected components containing two or more nodes and having three or more drug-resistant mutations are defined as “drug-resistant transmission clusters”.

### Statistical methods

Data entry and management were completed using Excel, and statistical analysis was performed with SPSS 23.0. Univariate logistic regression analysis was used for the preliminary screening of potential influencing factors. Variables(or any of their categorical strata) meeting the significance criterion of *P <*0.10 were included in a multivariate logistic regression model for in-depth analysis. All statistical tests in this study were two-sided, with *P <*0.05 used as the criterion for determining statistical significance.

## Results

### Characteristics of the study population

**Figure 1:**
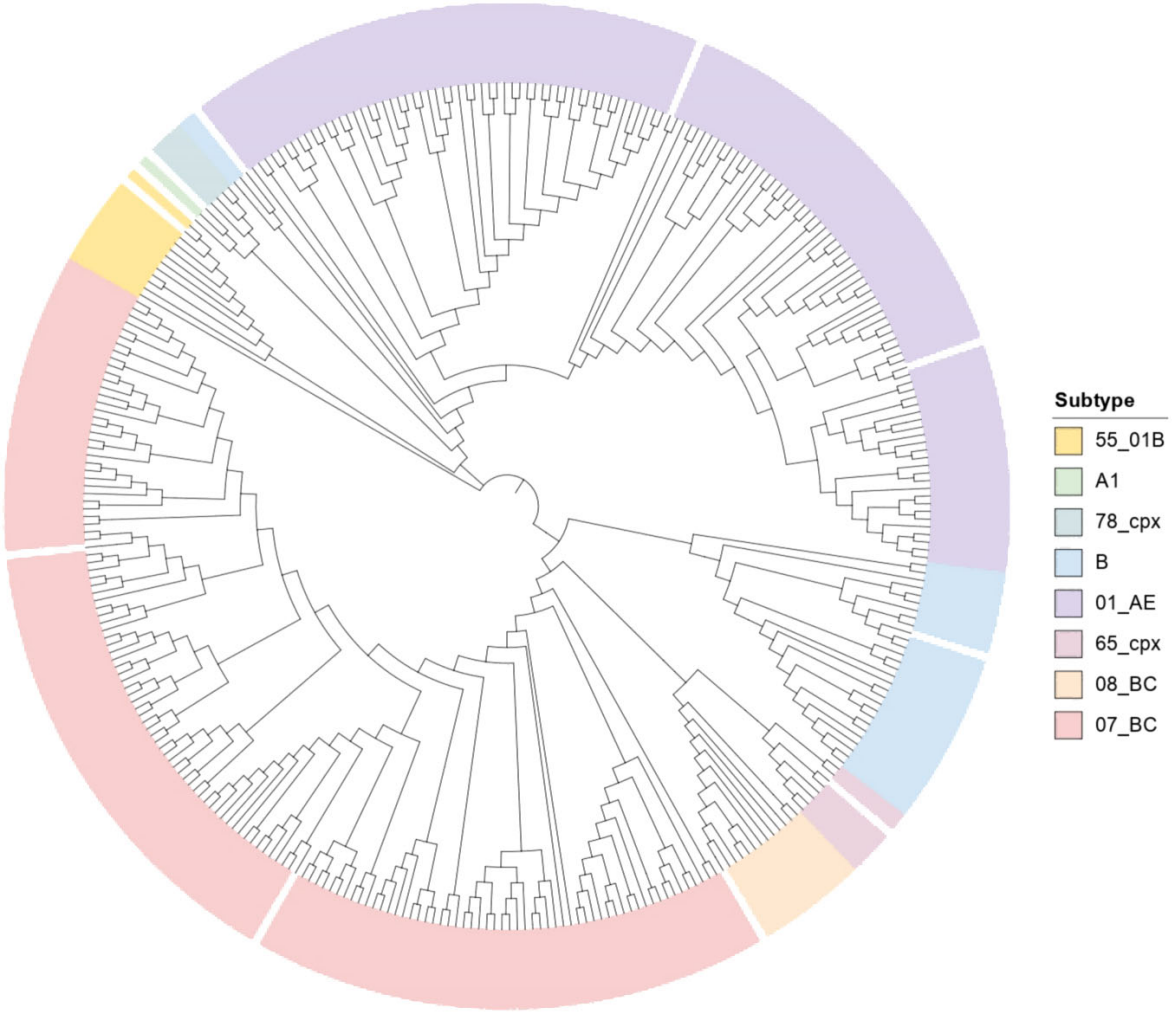
HIV-1 genotype distribution in the phylogenetic tree.

From January 2022 to June 2025, a consecutive sampling approach was employed to enroll 376 treatment-naïve, newly diagnosed cases aged ≥50 who were referred to the Hebei Provincial AIDS Confirmation Center. Among these comprehensively collected specimens, successful amplification of the *pol* gene sequences was achieved in 334 cases (88.8%). The resulting study population was predominantly male (80.2%), with a distinct concentration in the 50–59 age group (69.8%). Furthermore, most research subjects were of Han ethnicity (97.9%), and 77.8% were married. Farmers made up the largest occupational group (59.6%); contact between men who have sex with men (MSM) was the main transmission route (65.3%) (**Table 1**). After genotype analysis, a total of eight HIV-1 subtypes were detected, with main genotypes CRF07_BC (42.5%) and CRF01_AE (38.0%) and then subtype B (9.0%). Other subtypes included CRF55_01B, CRF08_BC, CRF65_cpx, CRF78_cpx, and subtype A1 (**Figure 1**).

**Table 1:** Sociodemographic characteristics of participants in Hebei Province, China.

Characteristics	Number	Percentage (%)
Gender		
Male	268	80.2
Female	66	19.8
Age (years)		
50–59	233	69.8
60–69	68	20.4
≥70	33	9.8
Marital status		
Unmarried	7	2.1
Married	260	77.8
Divorced/widowed	67	20.1
Ethnicity		
Han	327	97.9
Minority	7	2.1
Education level		
Primary or below	65	19.5
Junior high school	184	55.1
Senior high school	60	18.0
College or above	25	7.4
Occupation		
Farmers	199	59.6
Retirees	18	5.4
Commercial service	18	5.4
Others	99	29.6
Transmission route		
MSM	218	65.3
Heterosexual	116	34.7
Region		
Central Hebei	100	29.9
Southern Hebei	98	29.4
Eastern Hebei	105	31.4
Northern Hebei	31	9.3
Baseline CD4+ T-cell count (cells/µL)		
≤200	123	36.8
201–500	180	53.9
>500	31	9.3
Subtype		
Others^a^	35	10.5
CRF01_AE	127	38.0
CRF07_BC	142	42.5
B	30	9.0

^a^ Others include CRF55_01B, CRF08_BC, CRF65_cpx, CRF78_cpx, and subtype A1.

### Pretreatment drug resistance

An analysis of the test results revealed that the overall prevalence of PDR was 12.3% (41/334). Resistance to NNRTIs was the most common, accounting for 6.6% of the total cases. K103N and V179D were the predominant mutations. These two mutations are associated with high-level resistance to efavirenz (EFV) and nevirapine (NVP) and can mediate resistance to rilpivirine (RPV) and etravirine (ETR). Protease inhibitors (PIs) had a relatively low (5.1%) drug resistance rate. Nucleoside reverse transcriptase inhibitors (NRTIs) also had a low resistance rate at 2.7%. Seven MDR cases were detected: five with concurrent NRTI and NNRTI resistance and two with PI/NNRTI resistance (**Table 2**). These results show high treatment failure risk, requiring close patient monitoring.

**Table 2:** Distribution of DRMs in HIV-1 adults aged ≥50 in Hebei, China.

DRMs^a^	Number	PDR%	Drug^b^
Total	41	12.3	
NNRTIs	22	6.6	
K103N	4	1.2	EFV/ETR/NVP/RPV
V179D	4	1.2	EFV/ETR/NVP/RPV
K101E	2	0.6	EFV/ETR/RPV
E138G	1	0.3	EFV
G190S	1	0.3	DOR/EFV/ETR/RPV
K101H/N/Q	1	0.3	RPV
Y181C	1	0.3	EFV/ETR/NVP/RPV
V106A/I, V179V/D	2	0.6	DOR/EFV/ETR/NVP/RPV
V179D, M230M/L	1	0.3	DOR/EFV/ETR/NVP/RPV
L100I, Y188C	1	0.3	DOR/EFV/ETR/NVP/RPV
L100E/V, V106V/L, V179D	1	0.3	DOR/EFV/ETR/NVP/RPV
K103K/N, V106V/M, Y181Y/C	1	0.3	DOR/EFV/ETR/NVP/RPV
K101E, V106M, V179T, G190A, F227F/L	1	0.3	DOR/EFV/ETR/NVP/RPV
K101K/E, V106V/M, V179D, G190A, F227F/L	1	0.3	DOR/EFV/ETR/NVP/RPV
NRTIs	9	2.7	
M184M/V	4	1.2	ABC/FTC/3TC
M41L	1	0.3	AZT/D4T
M41L, S68S/G, M184V	1	0.3	ABC/DDI/FTC/3TC
K65R, S68G	2	0.6	ABC/D4T/DDI/FTC/3TC/TDF
D67G, Y115Y/F, M184V, K219N	1	0.3	ABC/AZT/D4T/DDI/FTC/3TC/TDF
PIs	17	5.1	
M46I	4	1.2	NFV
Q58E/Q58QE	7	2.1	TPV/r
K20T	2	0.6	NFV
L10L/F/F	2	0.6	FPV/r, NFV
I54IT	2	0.6	ATV/r, IDV/r, LPV/r, NFV, SQV/r, TPV/r

DOR, doravirine; ETR, etravirine; EFV, efavirenz; RPV, rilpivirine; NVP, nevirapine; DDI, didanosine; AZT, zidovudine; D4T, stavudine; FTC, emtricitabine; 3TC, lamivudine; ABC, abacavir; TDF, tenofovir disoproxil fumarate; NFV, nelfinavir; IDV, indinavir; SQV/r, saquinavir + ritonavir; TPV/r, tipranavir + ritonavir; ATV/r, atazanavir + ritonavir; LPV/r, lopinavir + ritonavir. ^a^Drug resistance mutations.

^b^ Drug indicates the 25 drugs available on the HIVDB database at Stanford University. Among the identified cases, seven exhibited multidrug resistance (MDR), including five cases with concurrent NRTI/NNRTI mutations (e.g., M184V + K103N) and two cases with PI/NNRTI mutations.

### HIV-1 molecular transmission network

We applied a genetic distance threshold of 1.5% for the network analysis. A total of 46 transmission clusters were identified, involving 117 gene sequences. About 35.0% of the sequences have close transmission linkage. Men accounted for 72.6% of the infected people in these clusters. Overall, 56.4% of the individuals in these clusters were infected through MSM contact. The transmission clusters vary among different viral subtypes. CRF07_BC formed the largest number of transmission clusters (22 in total), with a clustering rate of 43.7%, which was significantly higher than the 30.7% clustering rate of CRF01_AE. A nine-case CRF07_BC transmission cluster was found, with eight men aged 50–59, covering Central, Southern, and Eastern Hebei, showing cross-regional diffusion. A high-risk individual with a centrality of 5 in this cluster may be a key node in the transmission network. CRF01_AE forms a high-density subcluster structure. Its distribution concentrates in north-central Hebei Province.

### Factors associated with clustering

The results of the univariate analysis indicated a significant association between the transmission cluster status of HIV-infected individuals and factors such as gender, marital status, infection route, geographical location, and viral subtype (*P* < 0.05) (**Table 3**). Analysis based on the multivariate logistic regression model further identified two independent risk factors: divorce or widowhood (aOR = 2.040, 95% CI: 1.133–3.672, *P* = 0.018) and a history of living in Eastern Hebei (aOR = 2.401, 95% CI: 1.282–4.496, *P* = 0.006). In terms of viral subtypes, individuals infected with CRF01_AE (aOR = 0.539, *P* = 0.023) or other minor subtypes were less likely to appear in transmission clusters compared to those infected with CRF07_BC.

**Table 3:** Factors associated with clustering among older HIV-1 individuals in the networks.

Factors	Total (%) (*n* = 334)	Clustering (%) (*n* = 117; 35.0%)	Univariate analysis		Multivariate analysis	
			OR (95% CI)	*P*-value	aOR (95% CI)	*P*-value
Gender						
Male	268	85 (31.7)	1.00		1.00	
Female	66	32 (48.5)	2.026 (1.173–3.502)	0.011	1.750 (0.809–3.785)	0.155
Age (years)						
50–59	233	79 (33.9)	1.00			
60–69	68	25 (36.8)	1.133 (0.646–1.989)	0.663		
≥70	33	13 (39.4)	1.267 (0.599–2.680)	0.536		
Marital status						
Married	260	83 (31.9)	1.00		1.00	
Divorced/widowed	67	31 (46.3)	1.836 (1.163–3.172)	0.029	2.040 (1.133–3.672)	0.018
Unmarried	7	3 (42.9)	1.599 (0.350–7.309)	0.545	2.248 (0.460–10.984)	0.317
Ethnicity						
Han	327	116 (35.5)	1.00			
Minority	7	1 (14.3)	0.303 (0.036–2.549)	0.272		
Education level						
Primary or below	65	17 (26.2)	1.00			
Junior high school	184	69 (37.5)	1.694 (0.904–3.176)	0.100		
Senior high school	60	23 (38.3)	1.755 (0.821–3.751)	0.147		
College or above	25	8 (32.0)	1.329 (0.486–3.634)	0.580		
Occupation						
Farmers	199	63 (31.7)	1.00			
Retiree	18	9 (50.0)	2.159 (0.818–5.700)	0.120		
Commercial service	18	6 (33.3)	1.079 (0.387–3.007)	0.884		
Others	99	39 (39.4)	1.403 (0.850–2.317)	0.186		
Transmission route						
MSM	218	66 (30.3)	1.00		1.00	
Heterosexual	116	51 (44.0)	1.807 (1.133–2.882)	0.013	1.494 (0.776–2.878)	0.230
Baseline CD4+ T-cell count (cells/µL)						
≤200	123	44 (35.8)	1.00			
201–500	180	61 (33.9)	0.920 (0.569–1.489)	0.735		
>500	31	12 (38.7)	1.134 (0.504–2.552)	0.761		
Region						
Central Hebei	100	25 (25.0)	1.00		1.00	
Southern Hebei	98	37 (37.8)	1.820 (0.989–3.348)	0.054	1.852 (0.971–3.532)	0.061
Eastern Hebei	105	46 (43.8)	2.339 (1.291–4.239)	0.005	2.401 (1.282–4.496)	0.006
Northern Hebei	31	9 (29.0)	1.227 (0.500–3.012)	0.655	1.278 (0.497–3.288)	0.611
Subtype						
CRF07_BC	142	62 (43.7)	1.00		1.00	
CRF01_AE	127	39 (30.7)	0.572 (0.346–0.945)	0.029	0.539 (0.317–0.917)	0.023
B	30	12 (40.0)	0.860 (0.386–1.919)	0.713	0.702 (0.300–1.641)	0.414
Others	35	4 (11.4)	0.166 (0.056–0.497)	0.001	0.137 (0.045–0.420)	0.001

### Analysis of drug resistance transmission clusters

**Figure 2:**
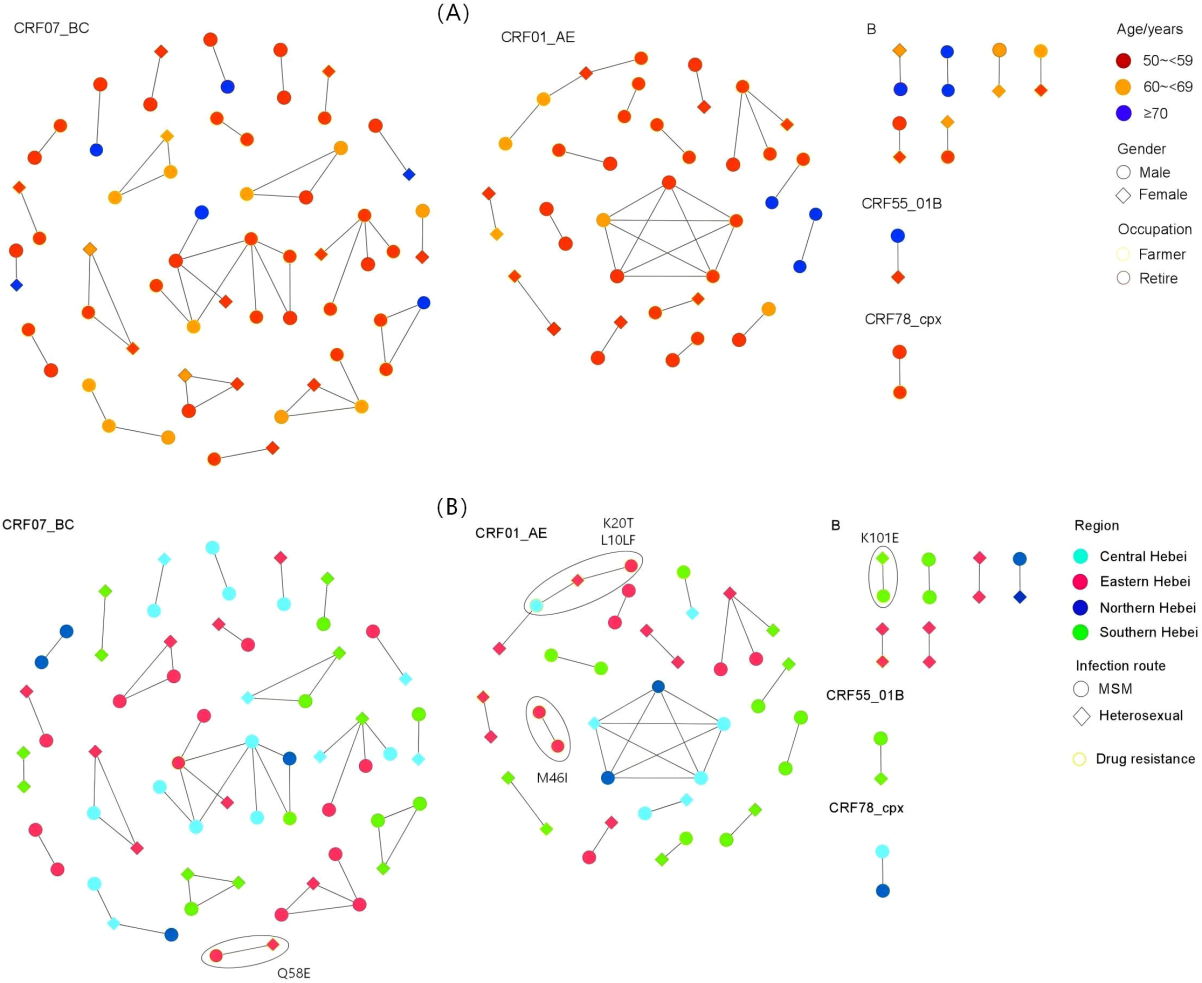
HIV-1 molecular transmission networks highlighting drug-resistant transmission clusters. **(A)** Transmission networks colored by age, gender, and occupation. **(B)** Transmission networks colored by geographical region, infection route, and drug resistance mutations.

This study identified nine drug resistance sequences in four transmission clusters. Analyzing the drug resistance characteristics of these clusters, it was found that protease-inhibitor-related mutations predominated (77.8%), and the specific mutations involved include Q58E, K20T, L10L/F, and M46I. Among them, a CRF01_AE transmission chain containing four cases was identified. This transmission cluster includes three cases with protease-inhibitor-associated mutations. Two carry K20T, and one has L10L/F, which weakens nelfinavir and fosamprenavir efficacy, showing low−level drug resistance. This result supports the association between the source of resistance and the initial infection event from an epidemiological perspective, further confirming that the strain is in a state of active transmission. In addition, an M46I mutation was detected in a smaller CRF01_AE cluster, which can cause moderate resistance to nelfinavir. We also observed that other subtypes showed regional clustering characteristics: there was a Q58E mutation in the CRF07_BC sequence in Eastern Hebei, which is associated with resistance to protease inhibitors. Meanwhile, a K101E mutation was found in the B subtype sequence in Southern Hebei, leading to cross-resistance to a variety of NNRTIs (**Figure 2**).

## Discussion

Our findings reveal a convergence of social vulnerability and biological risk in the aging HIV-1 epidemic in Hebei Province. Local PDR prevalence has reached 12.3%, exceeding the World Health Organization’s 10% warning threshold (World Health Organization, 2021). This phenomenon is primarily driven by NNRTI-associated mutations (6.6%), with K103N and V179D being the most common. Crucially, these mutations confer high-level resistance to first-generation NNRTIs, specifically EFV and NVP, and also cause varying degrees of cross-resistance to RPV and ETR. These results indicate that the standard ART regimens widely implemented in China, primarily based on EFV or NVP, carry a relatively high risk of virological failure in this specific older population. Older adults living with HIV often have age-related comorbidities and polypharmacy (Cruciani and Parisi, 2019), and they have a lower tolerance for treatment failure compared to younger cohorts. Therefore, the current NNRTI-based treatment strategies may no longer be suitable for older adults in this region.

Given the drug resistance characteristics of NNRTIs discovered in this study, it is necessary to consider transitioning to INSTIs, such as dolutegravir. This class of drugs has a relatively high genetic barrier to resistance and a lower risk of drug–drug interactions. However, it must be pointed out that our study sequenced only the *pol* region and did not include the integrase coding region. Therefore, baseline INSTI resistance profiles within this specific demographic remain uncharacterized. It is worth noting that we identified seven cases of MDR, accounting for 2.1% of the study population. Although the overall prevalence of MDR in China is still relatively low compared with single-class resistance, our findings are consistent with the increasingly complex trend of the drug resistance profiles observed in other regions such as Guangxi and Sichuan (Su et al., 2025; Pei et al., 2025). However, compared with the national average reported in a systematic review (<1%) (Zuo et al., 2020), the prevalence of PI-related mutations in our cohort (5.1%) was unusually high. This discrepancy suggests that older adults in Hebei Province may have distinct pathways of resistance accumulation, which may be related to their specific adherence challenges or transmission clusters involving resistant strains. Despite this, recent surveillance data from Hebei Province show that INSTI resistance (2.9%) among men who have sex with men (MSM) is on the rise (Lu et al., 2025). Given the observed active transmission clusters, we strongly recommend that baseline genotypic resistance testing for the integrase region be incorporated into routine clinical practice before initiating INSTI-based regimens.

The HIV molecular epidemiology of individuals aged ≥50 in this study looks quite different from what we typically see in the under−50 group. Regional data already show that in Hebei Province and several other parts of China, the epidemic among younger people—especially young MSM—is driven largely by CRF01_AE (Lu et al., 2017; Liu et al., 2025). Our findings add weight to a noticeable shift in subtype patterns once we cross the 50−year threshold: CRF07_BC made up 42.5% of cases in this older cohort, overtaking CRF01_AE as the dominant circulating strain. Recent domestic surveys that focused on younger infected individuals and general treatment−naïve populations have found PDR levels that are relatively low and rising only slowly, with prevalence falling between 4.7% and 7.4% (Zhang et al., 2024; Pang et al., 2023). In contrast, the PDR detection rate among the aged ≥50 participants in this study reached 12.3%—markedly higher than the figures reported3for those in other groups. The resistance mutations that we observed were largely clustered around NNRTI−related positions. These age−stratified differences in both epidemiology and resistance make one thing clear: the HIV−infected population aged ≥50 is not simply a subgroup being swept along by the broader epidemic. It has its own distinct patterns of transmission and viral evolution, along with a unique way of accumulating drug−resistant strains. This distinct subtype distribution aligns with the “dual-track” HIV epidemiological trends often observed across China. Recently, CRF07_BC has surpassed CRF01_AE as one of China’s most prevalent circulating recombinant lineages (Wang et al., 2024). The divergence between youth-dominated CRF01_AE and elderly-concentrated CRF07_BC epidemics indicates that older adults are not merely spillover targets but also likely constitute an independent, self-sustaining reservoir for the expansion of CRF07_BC. The high prevalence of CRF07_BC warrants close attention, and its virological characteristics pose special risks. Studies indicate that following infection with CRF07_BC, the viral load set-point of infected individuals is lower, and disease progression is slower (Huang et al., 2014; Li et al., 2014). There is a widespread problem of insufficient risk awareness of HIV infection and delayed diagnosis among older adults living with HIV. This seemingly “low pathogenicity” feature may enhance the transmissibility of the virus by prolonging the asymptomatic transmission period. This mechanism might have provided conditions for the formation of the large-scale hidden transmission chains discovered in this network analysis. On top of that, 36.8% of the participants had a baseline CD4^+^ T lymphocyte count of ≤200 cells/µL, which points to a fairly pronounced issue of diagnostic delay in this study population. Diagnostic delay brings with it a range of negative consequences. From an epidemiological angle, it stretches out the asymptomatic window in infected individuals, opening up more chances for the virus to spread under the radar. This likely helps explain the elevated proportion of clustered infections seen here, which came in at 35.0%. From a clinical standpoint, people with this degree of immune impairment have much less room to absorb a failure of first-line antiretroviral therapy. When you layer a transmitted drug resistance prevalence of 12.3% on top of that, the clinical stakes rise considerably, and the demands on treatment decisions become that much heavier.

A topological analysis of the transmission network shows that marital breakdown is an independent risk factor for infected individuals to be included in molecular transmission clusters. From the perspective of transmission dynamics, this indicates that older adults living with HIV without a fixed partner are engaging in highly close-contact sexual networks, where the rate of condom use is extremely low (Yuan et al., 2022). In addition, 77.8% of the infected individuals in this study were married, but 65.3% of the infection routes were attributed to MSM contact. Looking more closely, the cross-tabulation shows that 75.7% (165/218) of the individuals in our cohort who acquired HIV through MSM contact were currently married. This considerable overlap points to an important “bridging” role in transmission dynamics: married MSM can serve as a link connecting high-risk male sexual networks with the lower-risk general population of heterosexual partners. The risk of secondary spousal transmission is very real, and recent domestic work backs this up strongly. Phylogenetic analyses conducted in China, for instance, have confirmed cases where HIV-1 moved directly from married MSM to their heterosexual wives and children (Zhou et al., 2021). On top of that, traditional family values push a large majority of Chinese MSM toward heterosexual marriage eventually, which, in turn, leads to frequent unprotected sex within those marriages and leaves spouses at higher risk (Wu et al., 2020). Should a resistant strain find its way into these well-connected nodes of the network, it could move fast through both kinds of networks, making targeted interventions aimed at spouses a matter of real urgency. The research also identified a transmission cluster carrying protease inhibitor resistance mutations, which further confirmed the aforementioned transmission risk.

### Limitations

This study cannot clarify the direction of virus transmission within transmission clusters. A high proportion of MSM in the cohort may cause detection bias: older heterosexual adults have lower HIV screening standards and frequency than MSM, which may underestimate the burden of heterosexual HIV transmission. Additionally, reliance on self-reported transmission routes may introduce social desirability bias, leading to the potential underreporting of concurrent bisexual behaviors. Furthermore, our study exclusively included adults aged ≥50. Since older adults may also interact within sexual networks involving younger individuals (<50 years), the exclusion of younger patients from our phylogenetic analysis might underestimate the true transmission cluster frequency and overlook broader, intergenerational transmission chains, precluding us from performing further sensitivity analyses. Finally, observed regional clustering differences may be confounded by variations in local testing capacity and population size. Nevertheless, this study revealed a high transmission cluster formation rate and molecular evidence of PDR, holding reliable research value. Routine Sanger sequencing may fail to detect low-abundance drug-resistant variants, underestimating actual drug resistance prevalence. Future research should urgently employ next-generation sequencing to accurately identify such variants and analyze virus transmission direction.

## Conclusion

This study brings into focus the shifting dynamics of the HIV-1 epidemic among older adults in Hebei Province. The picture is marked by the dominance of CRF07_BC and a PDR prevalence of 12.3%, a figure driven largely by NNRTI mutations. The clustering rate stands at 35.0%, and it is clear that MSM contact and marital dissolution are pushing that number up. The fact that such a large share of participants are married MSM also raises serious concerns about hidden network growth and the risk of spouses getting infected secondarily. In light of these findings, moving toward INSTI-based antiretroviral regimens and rolling out targeted spousal testing and behavioral programs for older adults should be treated as pressing public health steps. Looking ahead, studies will need to tap into next-generation sequencing to get a sharper read on who is transmitting to whom and to catch low-abundance resistant variants that might otherwise slip by, details that matter for steering precision control efforts.

## Data Availability

The datasets presented in this study can be found in online repositories. The names of the repository/repositories and accession number(s) can be found below: https://www.ncbi.nlm.nih.gov/genbank/, PZ016112–PZ016445.
